# Returning to Work After Traumatic Spine Fractures: Current Status in a Military Hospital

**DOI:** 10.1093/milmed/usae012

**Published:** 2024-02-09

**Authors:** Abdulrahman Yousef Alhabeeb, Faisal Konbaz, Sami Aleissa, Ghada S Alhamed, Thamer S Alhowaish, Moustafa S Alhamadh, Emad Masuadi, Majed Abalkhail, Fahad AlHelal, Anouar Bourghli

**Affiliations:** College of Medicine, King Saud Bin Abdulaziz University for Health Sciences, Riyadh 11481, Saudi Arabia; King Abdullah International Medical Research Center, Riyadh 11481, Saudi Arabia; Ministry of the National Guard—Health Affairs, Riyadh 11426, Saudi Arabia; Department of Spine Surgery, King Faisal Specialist Hospital & Research Centre, Riyadh 11211, Saudi Arabia; Department of orthopedic surgery, Ministry of the National Guard—Health Affairs, Riyadh 11426, Saudi Arabia; Department of orthopedic surgery, Ministry of the National Guard—Health Affairs, Riyadh 11426, Saudi Arabia; College of Medicine, King Saud Bin Abdulaziz University for Health Sciences, Riyadh 11481, Saudi Arabia; King Abdullah International Medical Research Center, Riyadh 11481, Saudi Arabia; Ministry of the National Guard—Health Affairs, Riyadh 11426, Saudi Arabia; Division of Neurology, King Abdulaziz Medical City, Ministry of the National Guard Health Affairs, Riyadh, Saudi Arabia; College of Medicine, King Saud Bin Abdulaziz University for Health Sciences, Riyadh 11481, Saudi Arabia; King Abdullah International Medical Research Center, Riyadh 11481, Saudi Arabia; Ministry of the National Guard—Health Affairs, Riyadh 11426, Saudi Arabia; College of Medicine and Health science, United Arab Emirates University, United Arab Emirates; Department of orthopedic surgery, Ministry of the National Guard—Health Affairs, Riyadh 11426, Saudi Arabia; Department of orthopedic surgery, Ministry of the National Guard—Health Affairs, Riyadh 11426, Saudi Arabia; Department of Spine Surgery, King Faisal Specialist Hospital & Research Centre, Riyadh 11211, Saudi Arabia

## Abstract

**Introduction:**

The consequences of traumatic spine fracture (TSF) are complex and have a major burden on patients’ social life and financial status. In this study, we aimed to investigate the return to work (RTW) after surgically treated TSFs, develop eventual predictors of delayed or failure to RTW, and assess narcotics use following such injuries.

**Methods:**

This was a single-center retrospective cohort study that was performed in a tertiary care center. TSF patients who required surgical intervention from 2016 to 2021 were enrolled. Demographic, operative, and complication data, as well as narcotics use, were recorded. RTW was modeled using multivariate logistic regression analysis.

**Results:**

Within the 173 patients with TSF, male patients accounted for 82.7%, and motor vehicle accidents were the most common mechanism of injury (80.2%). Neurologically intact patients represented 59%. Only 38.15% returned to work after their injury. Majority of the patients didn’t use narcotics more than 1 week after discharge (93.1%). High surgical blood loss, operation time, and hospital length of stay were significantly associated with not returning to work. In multivariant regression analysis, every increase of 100 ml of surgical blood loss was found to decrease the chance of RTW by 25% (*P* = 0.04). Furthermore, every increase of one hour in operation time decreases the chance of RTW by 31% (*P* = 0.03).

**Conclusion:**

RTW is an important aspect that needs to be taken into consideration by health care providers. We found that age and high surgery time, blood loss, and hospital stay are significantly impacting patients’ RTW after operated TSF.

## INTRODUCTION

Traumatic spine fracture (TSF) has devastating consequences including pain, deformity, high mortality, and other psychological, economic, and social impact on the patients and their families.^1,2^ The causes of TSF vary based on the age, but most of which are attributed to motor vehicle accidents (MVAs), falls, and violence.^1,3^ Owing to its biomechanical weakness against external stress, thoracolumbar junction is most vulnerable to injury.^4^ Thoracolumbar fractures are considered the commonest spinal injury with an estimated annual incidence of more than 160,000 injuries in North America. It accounts for 50% to 75% of all spinal injuries, most of which occur at thoracolumbar junction, thoracic spine, and lumbar spine, accounting for 70%, 13%, and 18%, respectively.^2–5^

The psychological impact after the injury includes depression, anxiety, and posttraumatic stress disorder. Subsequently, it increases pain and substance abuse, and predicts poorer outcomes.^6,7^ Socially, patient with traumatic spinal cord injury can be affected greatly since these patients are usually young adults in their prime. Patients’ lives after the injury may be influenced by factors such as living independently, maintaining communication with friends and family, and returning to work.^8^ Moreover, SCI patients can be affected financially. Medications, physiotherapy, wheelchair, and lastly employment are important factors that SCI patients have to consider after the injury.^8–10^ Employment rates were assessed and found to be 62% for patients with disabilities compared to 78% in general population.^11^

Surgical fixation after unstable spinal fractures has been widely applied during the past decades as it proved to dramatically improve the patient’s recovery. Presumably, spinal surgery allows early fracture stabilization and patient mobilization, thus enhancing employment potential. However, neurological injury, polytrauma, and medical complications decrease functional independence, reducing the probability of employment after surgically treated spinal fracture.

Pain management is an important factor that determines the quality of life in patients with TSF. Narcotics can be used in pain management for neuropathic pain, but on the other hand, narcotics are considered very addictive. There is limited evidence describing the return to work (RTW) rates in patients who sustained a surgically treated spinal fracture in Saudi Arabia.

The primary objective of this study was to estimate the “return to work” in a 2-year follow-up after rehabilitation of patients with TSF, and secondary objectives include identifying its influencing factors and describing the use of narcotics in such population.

## METHODS

### Study Design and Aim

This was a single-center retrospective cohort study that was performed in a tertiary military care center in Riyadh, Saudi Arabia, to evaluate the employment status and narcotics use in TSF patients.

### Inclusion Criteria

All patients with TSF aged from 18 to 65 years old who required surgical intervention from 2016 until 2020 were included. Indications for surgical treatment included: three-column fractures or fracture-dislocations, fracture with neurological injuries or local deformity, unstable fractures in polytrauma patients.

### Data Collection

BestCare system, which is an electronic record system, was used to screen the electronic files and collect the data. Data related to patients’ demographics, comorbidities, injuries, operative complications, medications, length of stay, work status before and after the injury, duration to RTW, and change in the work or study field due to the injury were recorded. Early RTW was defined as RTW within 3 months and remaining at work for more than 6 months of the following year, and late RTW was defined as returning to work within 2 years and remaining at work for more than 6 months of the following year.

### Statistical Analysis

Statistical Package for the Social Sciences (SPSS version 22) was used for data analysis. Categorical variables were presented as frequencies and percentages whereas numerical variables were presented as mean ± standard deviation. Fisher’s Exact Test was used instead of Chi-square to test the association between categorical variables due to the relatively small sample size. To assess the predictors of not returning to work, multivariate logistic regression analysis was performed by calculating the adjusted odds ratios, and odds ratios were reported with 95% confidence interval. A test was considered significant if two-sided *P*-value <0.05.

### Ethical Considerations

Data collection commenced after obtaining the approval from the hospital research center (study number: NRC21R.124). Data were accessed by the research team only. Patient and subjects’ confidentiality and privacy were assured, no names or medical record numbers were used, and all data were kept in a secure place within the hospital premises.

## RESULTS

There were a total of 173 patients with TSF. The majority of the patients were males (82.7%) with a mean age of 33.8 ± 13.2 and an average Body mass index (BMI) of 25.44 ± 6.1. Only 26.3% of the patients were smokers (Table I). The mechanism of injury was MVA in 80.2% of the cases (Table I). Thoracolumbar injuries were found to be the most frequent injuries (70.5%). Three-column fractures were the most common fractures (32.2%) followed by fracture-dislocations (29.80%) (Table I). The most common associated injury was chest injury (41%) such as pneumothorax and rib fractures. Post-operative complications were infrequent with urinary tract infection being the commonest followed by venous thromboembolism, accounting for 9.2% and 5.2%, respectively. Intraoperatively, 20.2% of the patients required blood transfusion, and only 8% required postoperatively. The mean intraoperative blood loss was 338.47 mL ± 274, and the mean operation time was 226.4 minutes (3.7 hours). The mean length of stay in days was 44.89 days ± 76.3. Around half of the patients were admitted to the intensive care unit (44.51%) with a mean length of stay of 16.09 days ± 29.2 (Table II). After discharge, only 6.9% of the patients required continued narcotics in their follow-up appointments. Regarding the RTW, 38.15% of the patients have returned to their jobs after their injury with an average time to RTW of 8.9 months, and 24.24% of them had to change their work style, for example, desk job instead of field job, or had to change their university specialty due to the injury. Patients who returned to work early accounted for 9.25% (16). In regard to the American Spinal Injury Association (ASIA) score pre-operatively, majority of the patients (59.0%) were E (Table I). There was only 1.7% mortality.

**TABLE I.** Demographics

		*N*	%
Gender	Male	143	82.7
	Female	30	17.3
Age (years)	≤20	31	17.9
	21–30	55	31.8
	31–40	37	21.4
	41–50	22	12.7
	51–60	28	16.2
Body mass index	Underweight	15	8.8
	Normal	71	41.5
	Overweight	50	29.2
	Obese	35	20.5
Smoke	No	118	73.8
	Yes	42	26.3
Comorbidities	Diabetes mellitus	19	11
	Hypertension	20	11.6
	Dyslipidemia	12	6.9
ASA score	1	24	14.50
	2	67	40.40
	3	53	31.90
	4	21	12.70
	5	1	0.60
Mechanism of injury	MVA	138	80.2
	Fall from height	31	18.0
	Heavy on	2	1.2
	Blast	1	0.6
Level of fracture:	Upper cervical	9	5.20
	Lower cervical	40	23.10
	Thoracolumbar	122	70.50
	Pelvis and sacrum	2	1.20
Type of fracture	Dislocation	51	29.80
	Burst	49	28.70
	Chance (3 columns injuries)	55	32.20
	Compression	16	9.40
Pre-op neurological status	Intact	87	58.4
	Deficit	62	35.8
ASIA post-op	A	34	20.5
	B	4	2.4
	C	13	7.8
	D	17	10.2
	E	98	59.0 MVA, motor vehicle accidents.

**TABLE II.** Other Numerical Variables

	Minimum	Maximum	Mean	SD
Surgical blood loss in ml	0	2000	338.47	274.068
operation time in min	20	687	226.44	102.982
Length of stay	0	654	44.89	76.344
Postoperative hospital stay	3	230	19.83	39.311
ICU admission in days	1	245	16.09	29.263

Age was found to be significantly associated with returning to work, where younger patients tend to RTW more than older patients (*P*-value = 0.005). Type of fracture was also significantly associated with returning to work (*P*-value = 0.016). More than half (60%) of patients with burst fracture didn’t return to work. RTW was significantly associated with blood loss (*P*-value = 0.001), operation time (*P*-value = 0.003), length of stay (*P*-value = 0.012), and American Society of Anesthesiologists (ASA) score (*P*-value = 0.001). Patients with complete SCI (ASIA A) were associated significantly with RTW (*P*-value = 0.001). Gender, BMI, and mechanism of injury were not found to be significant predictors of RTW (*P*-value = 0.758, 0.098, and 0.084, respectively) (Table III).

**TABLE III.** Association between RTW with surgical blood loss, operation time, and ASA score.

	Got back to work	*N*	Mean	SD	*P*-value
Surgical blood loss	No Yes	47 66	447.0 264.4	369.9 180.6	0.001
Operation time	No Yes	44 66	265.52 205.5	105.362 100.959	0.003
ASA Score	No	44	2.75	0.967	0.001
	Yes	65	2.17	0.84	

Regarding the narcotics use, there was no statistically significant association of age and narcotic use (*P*-value = 0.061). Patients with SCI or chest-associated injuries tend to use more narcotics (*P*-value = 0.029). Smoking was significantly associated with narcotics use (*P*-value = 0.015), and we noted that smokers tended to use more narcotics.

Multivariant regression analysis showed an association between a 100 mL increase in blood loss during the operation and a 25% decrease in the likelihood of returning to work (odds ratio (OR) = 0.75, 95% confidence interval (CI) = 0.57–1, *P* = 0.04). Furthermore, each additional hour in operation time was associated with a 31% decrease in the chance of returning to work (OR = 0.69, 95% CI = 0.49–0.98, *P* = 0.03). Lastly, individuals under the age of 20 show a 20-fold higher likelihood of returning to work compared to those in the 50 to 60 age group (OR = 20.9, 95% CI = 3.2–133.9, *P* = 0.001), and a 10-fold higher likelihood compared to individuals aged 40 to 50 (OR = 21.1, 95% CI = 2.9–151.6, *P* = 0.002), indicating a notable association between younger age and increased probability of returning to work (Table IV).

**TABLE IV.** Multivariate Regression Analysis: Association between RTW with surgical blood loss, operation time, and age.

		*P*-value	OR	95% CI	
Surgical blood loss (100 ml)		0.047	0.75	0.57	1.00
operation time (hour)		0.038	0.69	0.49	0.98
Age (years)	≤20	0.001	20.98	3.29	133.99
	21–30	0.016	9.23	1.52	55.97
	31–40	0.002	21.14	2.95	151.63

## DISCUSSION

TSF patients tend to have the same profile, which is male in their third to sixth decade of life with the etiology being either MVA or falls from height. When comparing our results to previous studies done in Saudi Arabia, we can notice some similarities such as high male prevalence, mean age in the third decade, high thoracolumbar injuries, and MVA being the most common etiology.^12–14^ Compared to nearby countries like Kuwait, patients’ mean age, which is 37.1, and prominence of thoracolumbar injuries are similar features when compared to our study.^15^ Compared to worldwide data, thoracolumbar injury being the most prominent site of TSF is similar in Netherlands and China; however, the mechanism of injury is not, as it was found to be rather falls from height (44.7% and 58.9%, respectively, for both aforementioned countries).^16,17^

Traumatic spinal cord injury is tremendously devastating for the patients and their families due to the permanent or temporary limitations the injury carries. It can alter and disrupt patient’s quality of life in many ways, including dependency, chronic pain with use of narcotics, psychological damage, and economical damage in a form of losing/changing job or treatment costs in the short and long term.^1,2,6^ Independence is an important or arguably the most important aspect following traumatic SCI. It can influence the rehospitalization rate, psychological health, and employment. This burden is not limited to the patients but also to their families and care providers as those patients need special attention and care especially when neurologically affected or paraplegic.^18^

One of the main areas that can influence patients’ economical and psychological status is returning to work. While investigating whether patients are getting back to work or not after TSF, we found that only around one-third have returned to their work/education and one-fourth of them had to change their work or study field due to the injury. A review of the literature demonstrated employment can vary, and ranges from 13% up to 69%; similarly, in another review it ranged from 21% to 67%.^19^ In another study, they found among patients who were working before the injury, a few (9.1%) were self-employed. After the injury, 78.7% were still employed, and among those, 64.6% were self-employed. Moreover, only 11.48% have returned to the same jobs pre-injury. This raised an important issue that even if patients are returning to their jobs after the injury, they are not capable of doing the same previous job routine/performance.^20^ In a separate study conducted in Malaysia, the employment rate after injury stood at 76.2%.^21^ Contrasting this finding with existing literature, the notable disparity in return-to-work rates observed in our current study can be attributed to its focus on a trauma center, where patients presented with a range of injuries, including abdominal, brain, and lower limb injuries. Additionally, a considerable portion of our patient population comprised military personnel, whose occupational demands entail specific physical requirements. Notably, only 4 (2.3%) out of 173 patients underwent medical retirement.

Many patients, especially those aged over 40, who did not return to work chose to retire after their injury. Furthermore, in Saudi Arabia, individuals with disabilities can opt for retirement, regardless of their years of service, receiving a full salary. This retirement option was used by some patients also. For the factors that influence returning to work, the current study found that age is significantly associated with returning to work; likewise, many reviews and studies concluded the same association, younger patients tend to RTW more than older patients.

Burst fractures were found to be significantly negatively associated with returning to work, but there are no studies that investigated this association. This association could be explained by the severe nature of burst fractures that are often surgically managed when compared to other types such as simple compression fractures. Blood loss, operation time, and length of stay were found to be significantly associated factors with returning to work. In fact, longer OR time, blood loss, and length of stay may indicate more severe injuries, and therefore patients that would more likely have complications post-operatively. Furthermore, ASA score was significantly associated with returning to work. With higher ASA score, patients have more severe comorbidities that subsequently lead to poor prognosis and lesser chances of returning to work.

In a study by Burnham, it was found that 53.8% of the subjects returned to work after TSF and the most important predictor was whether the individual had worked within the year previous to injury (9.7 times more likely to be classified as employed at 1 year follow-up).^22^ In the current study, the majority of the patients were military personnel, and even if they were working before the injury, getting benefits from their status as military probably has led to the fact that more than 50% did not get back to their work.

After correcting for a number of important covariates, our logistic regression model found surgical blood loss, operation time, and hospital length of stay to be independent negative predictors of RTW. In a study by McLain, work status correlated directly with neurological impairment and was not related to level of injury, hardware failure, extent of surgical dissection, or construct pattern. However, only univariate analysis was used to determine those factors presented as independent predictors of outcome; a multivariate analysis would have been more appropriate to accurately establish the relation between the different variables.^23^

Opioid use for non-cancer pain has been steadily increasing over the past decades and became a serious public health concern.^24^ This is why it seemed important to assess narcotics use in the current study and try to understand the factors that lead to an increased consumption among the patients. There was no statistical significance between age and narcotic use, but it was noted that younger patients tend to use less narcotics compared to older patients. Moreover, smoking was found to be significantly associated with narcotics use. Many studies have found that patients who smoke cigarettes have higher frequency and doses of narcotics compared to non-smokers.

Surgeons should consider assessing and reassessing the socio-economic well-being of the patients throughout their recovery period. In a study investigating the priorities for physical and socioeconomic recovery after fractures of the extremities or the pelvis,^25^ it was shown that work-related recovery and disability benefits were a secondary concern compared with physical recovery in the first 12 months after the injury; however, the importance of work-related recovery was higher after the subacute phase.

In this study, we assessed the RTW in a Saudi population with TSFs and whether patients were able to continue the same work they had previously. To our knowledge, this is the first study to conduct such assessment in the Middle Eastern region. Furthermore, narcotics use in patients with spine injuries was evaluated which was not previously performed.

The limitations of this study include the fact that data were collected from a single military center. Next, its observational and retrospective design. The patients were not randomized for treatment and there were no controls; however, it is not possible to establish a randomized study in this type of patient cohort. There was a mixture of both neurologically intact and injured patients, but this should not revoke the value of the observed results. And finally the lack of a functional evaluation of the patients; however, this was not the objective of the current study. Multicentric studies will be required in the future to further investigate the factors related to RTW and narcotics use after TSFs in order to define specific preventive measures.

## CONCLUSION

Spinal fractures are not uncommon and the current study has highlighted the burden of unemployment or altered employment that often results. It was found that age, type of spinal fracture, surgical blood loss, operation time, and length of stay are significantly associated with patients RTW. The information may be useful for those responsible for vocational counseling in identifying patients at risk of unemployment so that proactive treatment strategies may be instituted.

## Data Availability

The data that support the findings of this study are available on request from the corresponding author.

## References

[1] Looby S, Flanders A: Spine trauma. Radiol Clin North Am 2011; 49(1): 129–63.doi: 10.1016/j.rcl.2010.07.019 21111133

[2] Ansar MN, Hashmi SM, Colombo F: Minimally Invasive Spine (MIS) surgery in traumatic thoracolumbar fractures: a single-center experience. Asian J Neurosurg 2020; 15(1): 76–82.doi: 10.4103/ajns.AJNS_236_19 32181177 PMC7057865

[3] Romero F, Vieira R, Ancheschi B: Open vs. percutaneous pedicle screw insertion for thoracolumbar traumatic A3 and A4 AO fractures - 18-months follow-up. Arquivos Brasileiros de Neurocirurgia: Brazilian Neurosurgery 2017; 36(04): 207–12.doi: 10.1055/s-0037-1607439.

[4] Spiegl UJ, Josten C, Devitt BM, Heyde CE: Incomplete burst fractures of the thoracolumbar spine: a review of literature. Eur Spine J 2017; 26(12): 3187–98.doi: 10.1007/s00586-017-5126-3 28547575

[5] McAnany SJ, Overley SC, Kim JS, Baird EO, Qureshi SA, Anderson PA: Open versus minimally invasive fixation techniques for thoracolumbar trauma: a meta-analysis. Global Spine J 2016; 6(2): 186–94.doi: 10.1055/s-0035-1554777 26933621 PMC4771513

[6] Migliorini C, Tonge B, Taleporos G: Spinal cord injury and mental health. Aust N Z J Psychiatry 2008; 42(4): 309–14.doi: 10.1080/00048670801886080 18330773

[7] Lim SW, Shiue YL, Ho CH, et al: Anxiety and depression in patients with traumatic spinal cord injury: a nationwide population-based cohort study. PloS One 2017; 12(1): e0169623.doi: 10.1371/journal.pone.0169623 PMC5231351 28081205

[8] Khazaeipour Z, Norouzi-Javidan A, Kaveh M, Khanzadeh Mehrabani F, Kazazi E, Emami-Razavi SH: Psychosocial outcomes following spinal cord injury in Iran. J Spinal Cord Med 2014; 37(3): 338–45.doi: 10.1179/2045772313Y.0000000174 24621045 PMC4064583

[9] Bennett J, Das JM, Emmady PD: Spinal cord injuries. In: *StatPearls*. StatPearls Publishing; 2022. 32809556

[10] Ahuja CS, Wilson JR, Nori S, et al: Traumatic spinal cord injury. Nat Rev Dis Primers 2017; 3(1): 17018.doi: 10.1038/nrdp.2017.18 28447605

[11] Holmlund L, Guidetti S, Eriksson G, Asaba E: Return-to-work: exploring professionals’ experiences of support for persons with spinal cord injury. Scand J Occup Ther 2021; 28(7): 571–81.doi: 10.1080/11038128.2020.1795245 32755475

[12] Konbaz F, AlEissa S, AlHabeeb A, et al: Consequences of neglected traumatic spinal cord injuries. J Taibah Univ Sci 2023; 18(2): 265–70.doi: 10.1016/j.jtumed.2022.09.017 PMC9926210 36817223

[13] Aldosari KH, Aldhfyan YM, Karrar MH, et al: Severity and neurosurgical management of patients with traumatic spinal fractures in Saudi Arabia: a cross sectional study. Pan Afr Med J 2019; 34(1): 26.doi: 10.11604/pamj.2019.34.26.19354 PMC6859040 31762894

[14] Alawad MO, Alenezi N, Alrashedan BS, et al: Traumatic spinal injuries in Saudi Arabia: a retrospective single-centre medical record review. BMJ Open 2020; 10(11): e039768.doi: 10.1136/bmjopen-2020-039768 PMC7668378 33191261

[15] Alhadhoud M, Alsiri N: The epidemiology of spinal fractures in a level 2 trauma center in Kuwait. SAGE Open Med 2021; 9: 20503121211051932.doi: 10.1177/20503121211051932 PMC8521410 34671474

[16] Wang H, Zhang Y, Xiang Q, et al: Epidemiology of traumatic spinal fractures: experience from medical university-affiliated hospitals in Chongqing, China, 2001-2010. J Neurosurg Spine 2012; 17(5): 459–68.doi: 10.3171/2012.8.SPINE111003 22978439

[17] den Ouden LP, Smits AJ, Stadhouder A, Feller R, Deunk J, Bloemers FW: Epidemiology of spinal fractures in a level one trauma center in the Netherlands: a 10 years review. Spine 2019; 44(10): 732–9.doi: 10.1097/BRS.0000000000002923 30395086

[18] Merritt CH, Taylor MA, Yelton CJ, Ray SK: Economic impact of traumatic spinal cord injuries in the United States. Neuroimmunol Neuroinflamm 2019; 6: 9.doi: 10.20517/2347-8659.2019.15 PMC8052100 33869674

[19] Lidal IB, Huynh TK, Biering-Sørensen F: Return to work following spinal cord injury: a review. Disabil Rehabil 2007; 29(17): 1341–75.doi: 10.1080/09638280701320839 17729082

[20] Ramakrishnan K, Mazlan M, Julia PE, Abdul Latif L: Return to work after spinal cord injury: factors related to time to first job. Spinal Cord 2011; 49(8): 924–7.doi: 10.1038/sc.2011.16 21383761

[21] Ramakrishnan K, Chung TY, Hasnan N, Abdullah SJ: Return to work after spinal cord injury in Malaysia. Spinal Cord 2011; 49(7): 812–6.doi: 10.1038/sc.2010.186 21221119

[22] Burnham RS, Warren SA, Saboe LA, Davis LA, Russell GG, Reid DC: Factors predicting employment 1 year after traumatic spine fracture. Spine 1996; 21(9): 1066–71.doi: 10.1097/00007632-199605010-00015 8724091

[23] McLain RF : Functional outcomes after surgery for spinal fractures: return to work and activity. Spine 2004; 29(4): 470–Z6.doi: 10.1097/01.brs.0000092373.57039.fc 15094545

[24] Zgierska A, Wallace ML, Burzinski CA, Cox J, Backonja M: Pharmacological and toxicological profile of opioid-treated, chronic low back pain patients entering a mindfulness intervention randomized controlled trial. J Opioid Manag 2014; 10(5): 323–35.doi: 10.5055/jom.2014.0222 25350474 PMC4321804

[25] O’Hara NN, Kringos DS, Slobogean GP, Degani Y, Klazinga NS: Patients place more of an emphasis on physical recovery than return to work or financial recovery. Clin Orthopaedics Related Res 2021; 479(6): 1333–43.doi: 10.1097/CORR.0000000000001583 PMC8133069 33239518

